# Evaluation of Renal Artery Variations in Horseshoe Kidneys with Computed Tomography

**DOI:** 10.5152/tud.2023.22222

**Published:** 2023-03-01

**Authors:** Azad Hekimoglu, Onur Ergun, Erdem Birgi, Aynur Turan, Baki Hekimoglu

**Affiliations:** Department of Radiology, Dışkapı Yıldırım Beyazıt Training and Research Hospital, Ankara, Turkey

**Keywords:** Horseshoe kidney, renal artery, variation, computed tomography

## Abstract

**Objective:**

Horseshoe kidney is the most common renal fusion anomaly, accounting for 90%. The study aims to explore the variations in the renal arteries of individuals with horseshoe kidney anomalies and contribute to the classification in the literature.

**Materials and methods:**

Computed tomography images of 145 individuals who had intravenous contrast-enhanced abdominal computed tomography for any reason and had horseshoe kidney anomalies were analyzed retrospectively, and the presence, origin, and number of accessory renal arteries were evaluated. Then, classification was performed according to the origin of the accessory arteries.

**Results:**

In 145 individuals, 44 different combinations of the accessory artery according to the origin and number were obtained. Most common accessory artery combination was type 2a (M1). According to our classification, 13.1% of the patients were type 1, 57.2% were type 2, 17.2% were type 3, 10.3% were type 4, and 1.4% were type 5.

**Conclusion:**

The classifications of arterial variations in horseshoe kidney anomalies did not match each other in previous studies and did not comprise all patients because they were conducted with a small number of cases. A more comprehensive new classification was created in our study according to accessory artery origins with the help of previous studies.

**Main Points**

- The incidence of renal artery anomalies in horseshoe kidneys is significantly high.
- In our study, 44 different combinations were obtained according to the origin and number of accessory arteries in 145 people.
- A new classification for arterial variations in horseshoe kidneys would be useful to reduce complications during surgeries or interventional procedures.

## Introduction

There are many studies on vascular variations of the kidneys.^1,2^ The increase in renal surgery and interventional procedures, especially in renal transplantations, has led to more studies on this subject. However, there are not enough studies investigating renal vascular variations in patients with congenital kidney anomalies such as horseshoe kidneys. Horseshoe kidney is the most common renal fusion anomaly, accounting for 90% of all renal anomalies, and has a prevalence of approximately 0.25% in the population.^3^ The studies performed were generally with cadavers, and classification was made for horseshoe kidney vascularization with 13 patients by Graves and 14 patients by Boatman et al.^4,5^ The first study performed with computed tomography (CT) angiography was by Ichikawa et al.^6^ In this study, renal arterial classification was executed in horseshoe kidneys with 39 patients, and the incidence of renal arterial variations in horseshoe kidneys was evaluated. 

This study aims to investigate renal arterial variations in individuals with horseshoe kidney anomalies who underwent abdominal CT for any reason and to bring a more comprehensive classification into the literature, including new types that do not fit the current classification.

## Materials and Methods

Images of 154 patients with renal anomaly who underwent abdominal CT with intravenous contrast for any reason in our hospital were analyzed retrospectively. Four of the patients examined were excluded from the study because they had a fusion anomaly other than the horseshoe kidney anomaly called crossed fused ectopia. Also, 5 patients were not included in the study, although horseshoe kidney anomaly was observed since CT images were insufficient for vascular evaluation. The mean age of 145 patients included in the study was 49.51 (age range: 19-90 years), of which 116 were men (80%) and 29 were women (20%).

**Figure 1.**
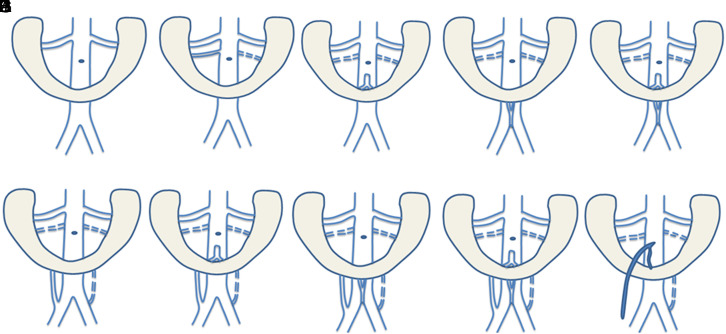
Schematic view of horseshoe kidney types; (A) Type 1a-no accessory arteries; (B) Type 1b-accessory artery originating from the lateral aorta; (C) Type 2a-originating of the accessory artery as the median artery from the anterior of the aorta;(D). Type 2b-originating accessory artery from the sacral artery; (E) Type 2c-accessory arteries originating from both the median artery and the sacral artery; (F) Type 3- presence of an accessory artery originating from the iliac artery; (G)Type 4a- presence of accessory arteries originating from the median and common iliac arteries; (H)Type 4b-presence of accessory arteries originating from the sacral and common iliac arteries; (I) Type 4c-accessory arteries originating from median, sacral and common iliac arteries; (J)Tip 5- presence of accessory artery originating from IMA. Dashed lines indicate the presence of an accessory artery. IMA, inferior mesenteric artery.

Abdominal CTs of the patients were performed with a 128-slice multidetector CT scanner (GE, Optima CT 660). Computed tomography images were taken with the parameters of 120 kV, 60-100 mA, 0.625 mm section thickness. Thin-section source images obtained in the axial plane were recalled from Picture Archiving and Communication Systems (PACS) and examined retrospectively in all patients. Multiplanar reformatted (MPR) images and maximum intensity projection (MIP) images were obtained from all patients. Three radiologists with at least 5 years of experience in cross-sectional imaging evaluated all CT images and agreed on their accuracy. Evaluations were made on MPR and MIP images in the axial and coronal planes. The presence, origin, and number of accessory renal arteries in both kidneys in the axial and coronal planes were evaluated. Then, classification was done according to the origin of the accessory arteries (Table 1 and Figure 1).

This study has been approved by the Ethics Committee of the Dışkapı Yıldırım Beyazıt Training and Research Hospital (protocol number: 121/11-2021). All methods were performed in accordance with the relevant guidelines and regulations and informed consent from all the patients has been taken.

## Results

The accessory renal arterial structure of all patients included in the study is shown in Table 2. According to the origin and number of accessory arteries of 145 patients, 44 different combinations were obtained. The most common accessory artery combination is type 2a (M1), and the number of patients with this combination was 23 (16%). The second most common combination was type 2c (M1, S1) with 14 (10%) patients and type 2b (S1) with 13 (9%) patients as the third most common combination. Other combinations were lower in number. In particular, type 3 and type 4 consisted of very different combinations. The combination with the highest number of accessory arteries was the combination of type 2c (R2; L1; M2; S1) with 6 accessory arteries. There were only 4 patients with no accessory artery (type 1a).

**Figure 2.**
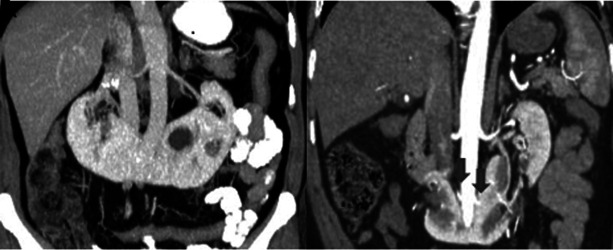
Type 1 accessory artery. (A)Type 1a; (B) Type 1b.

**Figure 3.**
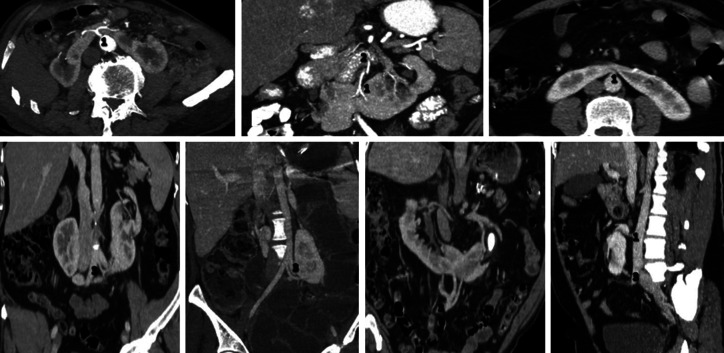
Type 2 accessory artery. (A) Median artery supplying the isthmus of the horseshoe kidney on axial CT image. (B) Median artery supplying the isthmus of the horseshoe kidney on coronal CT image. (C) Median artery supplying the isthmus of the horseshoe kidney on axial CT image. (D, E) Accessory artery originating from the sacral artery feeding the isthmus of the horseshoe kidney on coronal CT image. (F) Accessory artery originating from the sacral artery divides into several branches distally and feeds the isthmus of the horseshoe kidney and the inferior parts of both kidneys on coronal CT image. (G) Accessory arteries originating from both the median and sacral arteries on sagittal CT image.1*- *Accessory artery originating from the median artery; 2-IMA; 3- accessory artery originating from the sacral artery. IMA, inferior mesenteric artery; CT, computed tomography.

**Figure 4.**
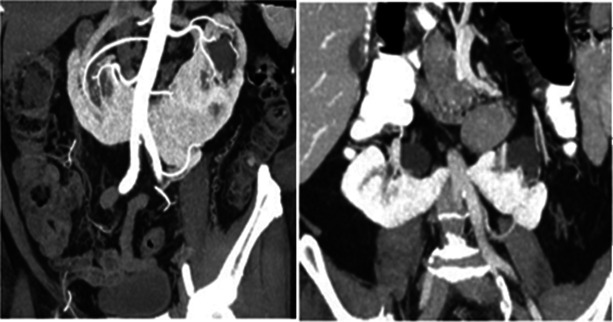
Type 3 accessory artery.

**Figure 5.**
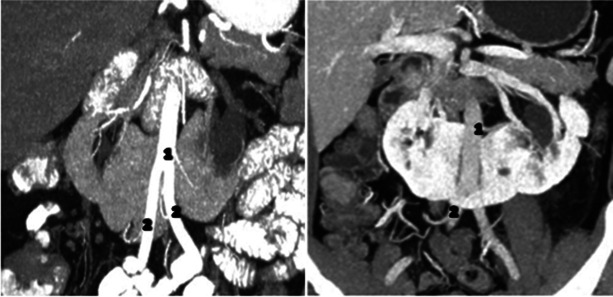
Type 4 accessory artery 1-accessory artery originating from the median artery; 2-accessory artery originating from the sacral artery.

**Figure 6.**
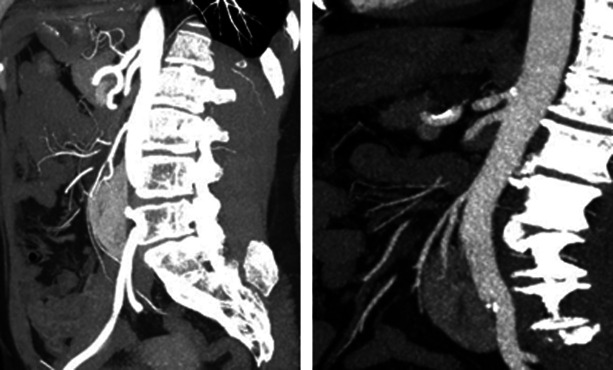
Type 5 accessory artery.

According to our classification, 19 (13.1%) of the 145 patients included in the study were type 1, of which 4 (2.8%) were type 1a and 15 (10.3%) were type 1b (Figures 1 and 2). Out of the 84 (57.9%) patients who were type 2, 35 (24.1%) were type 2a, 32 (22.1%) were type 2b, and 17 (11.7%) were type 2c (Figures 1 and 3). Type 3 was seen in 25 (17.2%) patients (Figures 1 and 4). Out of the 15 (10.3%) patients who were type 4, 13 (9%) were type 4a, 1 (0.7%) was type 4b, and 1 (0.7%) was type 4c (Figures 1 and 5). Lastly, 2 (1.4%) of the patients were type 5 (Figures 1 and 6).

## Discussion

Horseshoe kidney is the most common renal fusion anomaly, combining 3 anatomical abnormalities, which are ectopia, malrotation, and vascular changes, and in most cases, the abnormality consists of 2 renal tissues fused at their lower pole with a parenchymal or fibrous isthmus. Horseshoe kidney results from the fusion of metanephric buds between fourth and eighth weeks of embryogenesis, preventing cephalic migration and normal rotation of the buds. The inferior mesenteric artery prevents cranial migration of the isthmus of the fused kidney in the lower abdomen.^7,8^ The main renal arteries usually develop as normal. In addition, the mesonephrogenic arteries often continue to vascularize the upper part of the kidney, while the lower segmental metanephric arteries supply blood to the lower part of the kidney.^8^ This anomaly is found more frequently in men than in women at a ratio of 2 : 1.^9^ Yet, the incidence rate in men compared to women was 4 : 1 in our study.

A typical horseshoe kidney shows great variation in the origin, number, and size of arteries due to the retention of primitive veins.^10^ Majos et al^11^ revealed that the characteristic of the horseshoe kidney vascular system is the widespread presence of accessory renal arteries and the high frequency of renal arteries that avoid the renal hilus and enter the kidney directly from the parenchyma. When Table 2, which includes the accessory artery combinations of all our patients, is examined, it is seen how much arterial variation is seen in horseshoe kidney patients, with 44 combinations according to the origin and number of accessory arteries in 145 patients. Having so many variations makes classification difficult. While there are already few studies in the literature trying to classify arterial variations in horseshoe kidneys, the existing studies are insufficient in terms of the number of patients. According to some authors, a simple classification system is not possible due to the wide variations in the arterial pattern of the horseshoe kidneys reported in the literature.^8^ Therefore, classification in our study was done according to the origin of the accessory arteries, not the number. We thought that classification would be very complex and complicated if we included the number of accessory arteries. Since the combinations of accessory arteries are too many and different, we think it would be more explanatory to write the number and origin of all accessory arteries in parentheses beside the variation type (Table 2).

Classification of arterial variations in horseshoe kidneys by Ichikawa et al^6^ with CT and by Graves^4^ with cadaver has been done with a small number of cases. These classifications are inconsistent with each other and do not cover all patients. Our study aimed to create a new classification by making use out of these 2 classifications. In our classification, in the group we evaluated as type 1, the accessory artery was either absent or originated from both sides of the abdominal aorta. In addition to type 1, it was named type 2 when the accessory artery originates from the anterior of the abdominal aorta. It was named type 2a in the presence of an accessory artery originating from the anterior of the abdominal aorta and called the median branch, type 2b in the presence of an accessory artery originating from the sacral artery and type 2c if both were present. In addition to type 1, in the presence of accessory arteries originating from the common iliac arteries, the patients were evaluated as type 3. The classification done so far has been somewhat compatible with Graves’ classification. Patients who have type 2 and type 3 together were evaluated as type 4. As in type 2, if there is an accessory branch median artery originating from the anterior as a single branch, it was named type 4a. If there is an accessory artery originating from the sacral artery, it was named type 4b, and if both exist together, it was named type 4c. Lastly, we had 2 patients that we evaluated as type 5. These 2 patients had accessory branches from the inferior mesenteric artery (IMA), feeding the kidney. Arterial branch feeding the kidney with IMA branches has never been encountered in the literature, and we contributed it to the literature for the first time with images of 2 patients in our study (Figure 6). In addition, due to accessory arteries having many and different combinations, the initial letter and number of the origin of all accessory arteries were indicated in parentheses, as well as the type of variation.

In Papin’s^12^ autopsy study, cases with normal renal arteries constituted 20% of all horseshoe kidneys, those with 3-5 accessory arteries accounted for 66% of all cases, and those with more than 5 accessory arteries constituted 14% of all cases. In our study, the number of patients with no detected accessory artery was only 4 (2.8%). The most common type in our study was type 2 (57.9%), and the most common combination was type 2a (M1). The second most common type was type 2c (M1, S1), and the third most common was type 2b (S1). This may mean that isthmus blood supply is preferred with median and sacral arteries in horseshoe kidneys. In our study, except for the native renal arteries, the highest number of accessory arteries was 6, and it was seen in type 2c (R2; L1; M2; S1) combination, and in 2 patients, with 5 accessory arteries and seen in type 4a (R1a,1i; L1a,1i; M1) and type 4a (R1a; L2a,1i; M1) combinations. The number of accessory arteries in the other patients ranged from 1 to 4, excluding the native arteries.

Horseshoe kidney in adults is usually an asymptomatic disease, and its detection during intravenous pyelography, routine ultrasound, or computed tomography scan performed for other reasons is incidental.^5^ Pathological conditions such as renal stones, pelviureteric junction obstruction, hydronephrosis, and recurrent infections are common in horseshoe kidneys, and it is estimated that 25% of horseshoe kidneys require operative treatment.^5,9,13^ Especially, vascular abnormalities are of great importance in horseshoe kidney surgery. Multiple accessory arteries found in most horseshoe kidneys may endanger the life of patients intraoperatively due to unexpected bleeding.^9^ A study found that 0.12% of patients treated for abdominal aortic aneurysm had horseshoe kidneys.^14^ Kaplan et al^15^ reported that half of the patients who underwent endovascular aortic repair (EVAR) had small postoperative infarctions. They also suggest that patients with abdominal aortic aneurysm and horseshoe kidneys who are considered for EVAR should have accessory renal arteries with a maximum diameter of less than 3 mm and no evidence of prior renal failure.^15^ Majos et al^11^ looked at the level of origin of the renal arteries in horseshoe kidneys, emphasizing that the renal arteries branch significantly below the origin of the inferior mesenteric artery in horseshoe kidneys and that such vessels are usually smaller than those branching above this topographic mark. However, we think that the criteria of being under the origin of the IMA are not valid for all patients. As can be seen in Figure 3f, we see that the artery originating from the sacral artery has multiple distal branches, feeding not only the isthmus but also the inferior parts of both kidneys very widely. We think that such accessory arteries should be considered in interventions such as surgery or EVAR. It is very important since accessory arteries are tied during open surgery of abdominal aortic aneurysms or covered with a graft during endovascular procedures. In addition, bypass treatment and angioplasty of the iliac vessels may result in obliteration of the extrarenal arteries branching at this level.

The only considerable limitation of our study may be that we could not make a classification according to the number of accessory arteries, as we thought it might be very complex and complicated.

In conclusion, renal artery anomaly incidence in horseshoe kidneys is significantly higher than in a normal kidney. Detection of renal artery anomalies by CT before surgery or interventional procedure is necessary to reduce potential complications. Although our study has the highest number of patients on this subject in the literature, we think that series with an even higher number of patients are needed due to the wide variety of arterial variations in horseshoe kidneys.

## Figures and Tables

**Table 1.** Classification of Arterial Variations in Horseshoe Kidneys

Type 1	The accessory artery was either absent or originating from both sides of the abdominal aorta (Figures 1 and 2).
1a	There is no accessory artery.
1b	Accessory arteries originate from both sides of the abdominal aorta.
Type 2	In addition to type 1, the accessory artery originates from the anterior of the abdominal aorta (median) or sacral arteries (Figures 1 and 3).
2a	Accessory arteries originate from the anterior of the abdominal aorta as the median artery.
2b	The accessory artery originates from the sacral artery.
2c	Coexistence of median and sacral accessory arteries.
Type 3	In addition to type 1, in the presence of accessory arteries originating from the common iliac arteries (Figures 1 and 4).
Type 4	Accessory arteries arise both from the anterior of the abdominal aorta as median or sacral accessory arteries and from the common iliac arteries (Figures 1 and 5).
4a	Coexistence of median accessory artery and iliac artery.
4b	Coexistence of sacral accessory artery and iliac artery.
4c	Coexistence of median, sacral and iliac accessory artery.
Type 5	Presence of accessory artery originating from IMA (Figures 1 and 6).

IMA, inferior mesenteric artery.

**Table 2.** Types of All Patients and Combinations of Accessory Arteries

Type 1b (R1; L1) Type 1b (R1; L1) Type 1b (R2; L2) Type 1b (R1; L1) Type 1a Type 1b (R1) Type 1a Type 1a Type 1b (R1; L1) Type 1b (R1; L1) Type 1b (R1; L1) Type 1b (R1; L1) Type 1b (R1; L1) Type 1a Type 1b (R1; L1) Type 1b (R1; L1) Type 1b (R1; L1) Type 1b (R1) Type 1b (R1) Type 2a (M2) Type 2a (L1; S1) Type 2a (R1; L1; M1) Type 2b (S1) Type 2c (M1; S1) Type 2a (M1) Type 2b (R1; S1) Type 2b (R1; L1; S1) Type 2b (R1; L1; S1) Type 2b (S1)	Type 2b (R1; L1; S1) Type 2b (R1; L1; M1) Type 2c (M1; S1) Type 2b (S1) Type 2a (M1) Type 2a (M1) Type 2b (S1) Type 2b (S1) Type 2a (M1) Type 2b (S1) Type 2a (M1) Type 2c (R1; L1; M1; S1) Type 2a (M1) Type 2b (L1, S1) Type 2b (S1) Type 2b (R1; L1; S1) Type 2b (S1) Type 2c (M1; S1) Type 2a (M1) Type 2a (M1) Type 2b (R2; L1; S1) Type 2a (M1) Type 2a (M1) Type 2c (M1, S1) Type 2c (R2; L1; M2; S1) Type 2a (R1; L1; M1) Type 2a (L1, M1) Type 2a (M1) Type 2a (M1)	Type 2b (L1; S1) Type 2a (M1) Type 2b (S1) Type 2a (M1) Type 2a (R1; L1; M2) Type 2a (L1; M1) Type 2a (R1; M1) Type 2c (M1; S1) Type 2a (M1) Type 2a (M1) Type 2b (R1; S1) Type 2b (R1; L1; S1) Type 2b (R1; L1; S1) Type 2b (S1) Type 2a (M1) Type 2b (R1; L1; S1) Type 2a (M1) Type 2c (M2, S1) Type 2a (M2) Type 2a (R1; L1; M1) Type 2b (R1; L1; S1) Type 2a (M1) Type 2c (M1; S1) Type 2c (M1; S1) Type 2b (S1) Type 2a (M2) Type 2b (R2; L1; S1) Type 2b (L1; S1) Type 2b (R1; L1; S1)	Type 2a (M1) Type 2c (M1; S1) Type 2c (M1; S1) Type 2c (M1; S1) Type 2c (M1; S1) Type 2a (M1) Type 2b (R1; L1; S1) Type 2b (S1) Type 2b (S1) Type 2b (L1; S1) Type 2c (M1; S1) Type 2c (M1; S1) Type 2a (M2) Type 2c (M1; S1) Type 2a (M1) Type 2a (M1) Type 3 (R1a,1i; L1a,1i) Type 3 (R1i; L1i) Type 3 (R1a, 1i) Type 3 (R1a; L1a,1i) Type 3 (R1a,1i; L1a,1i) Type 3 (R1i; L1a,1i) Type 3 (R1a,1i; L1a,1i) Type 3 (R1a; L2i) Type 3 (R1a,1i; L1a,1i) Type 3 (R2i; L1i) Type 3 (R1a; L1i) Type 3 (R1a,1i; L1a,1i) Type 3 (R1i; L1a)	Type 3 (R1a; L1i) Type 3 (R1i; L1i) Type 3 (R1a,1i; L1i) Type 3 (R1a,1i; L1a,1i) Type 3 (R1a,1i; L1a,1i) Type 3 (R1a,1i; L1a) Type 3 (R1i; L1i) Type 3 (R2i; L1a) Type 3 (R2a; L1a,1i) Type 3 (R1a,1i; L1a,1i) Type 3 (R1a,1i) Type 3 (R1i; L1i) Type 4a (R1i; L1i; M1) Type 4a (R1a; L2a,1i; M1) Type 4a (L1a,1i; M1) Type 4a (R1i; L1i; M2) Type 4c (R1i; M1; S1) Type 4a (R1a,1i; L1a; M1) Type 4a (R1a,1i; L1a,1i; M1) Type 4a (R1i; M1) Type 4a (R1i; L2a; M1) Type 4a (R1i; L1i; M1) Type 4a (R1i; M1) Type 4a (R1i; L1i; M1) Type 4a (R1a,1i; L1a; M1) Type 4a (R1i; L1i; M1) Type 4b (R1a,1i; L1a; S1) Type 5 (R1a,1i; IMA1) Type 5 (M1; IMA 1)

R, right; L, left; a, aortic origin; i, iliac artery origin; M, originating from the median artery; S, originating from the sacral artery; IMA, originating from the inferior mesenteric artery (the number next to each letter indicates the number of accessory arteries.)
